# Prognostication of Pancreatic Cancer Using The Cancer Genome Atlas Based Ferroptosis-Related Long Non-Coding RNAs

**DOI:** 10.3389/fgene.2022.838021

**Published:** 2022-02-14

**Authors:** Jiayu Li, Jinghui Zhang, Shuiliang Tao, Jiaze Hong, Yuyan Zhang, Weiyan Chen

**Affiliations:** ^1^ The Second School of Clinical Medicine, Zhejiang Chinese Medical University, Hangzhou, China; ^2^ School of Life Sciences, Zhejiang Chinese Medical University, Hangzhou, China; ^3^ School of Basic Medical Sciences, Zhejiang Chinese Medical University, Hangzhou, China

**Keywords:** pancreatic adenocarcinoma, ferroptosis, long non-coding RNA, risk model, nomogram

## Abstract

**Background:** Long non-coding RNAs (lncRNAs) are key regulators of pancreatic cancer development and are involved in ferroptosis regulation. LncRNA transcript levels serve as a prognostic factor for pancreatic cancer. Therefore, identifying ferroptosis-related lncRNAs (FRLs) with prognostic value in pancreatic cancer is critical.

**Methods:** In this study, FRLs were identified by combining The Cancer Genome Atlas (TCGA) and FerrDb databases. For training cohort, univariate Cox, Lasso, and multivariate Cox regression analyses were applied to identify prognosis FRLs and then construct a prognostic FRLs signature. Testing cohort and entire cohort were applied to validate the prognostic signature. Moreover, the nomogram was performed to predict prognosis at different clinicopathological stages and risk scores. A co-expression network with 76 lncRNA-mRNA targets was constructed.

**Results:** Univariate Cox analysis was performed to analyze the prognostic value of 193 lncRNAs. Furthermore, the least absolute shrinkage and selection operator and the multivariate Cox analysis were used to assess the prognostic value of these ferroptosis-related lncRNAs. A prognostic risk model, of six lncRNAs, including LINC01705, AC068620.2, TRAF3IP2-AS1, AC092171.2, AC099850.3, and MIR193BHG was constructed. The Kaplan Meier (KM) and time-related receiver operating characteristic (ROC) curve analysis were performed to calculate overall survival and compare high- and low-risk groups. There was also a significant difference in survival time between the high-risk and low-risk groups for the testing cohort and the entire cohort, with AUCs of .723, .753, respectively. Combined with clinicopathological characteristics, the risk model was validated as a new independent prognostic factor for pancreatic adenocarcinoma through univariate and multivariate Cox regression. Moreover, a nomogram showed good prediction.

**Conclusion:** The signature of six FRLs had significant prognostic value for pancreatic adenocarcinoma. They may be a promising therapeutic target in clinical practice.

## 1 Introduction

Pancreatic adenocarcinoma (PAAD) is an aggressive gastrointestinal malignancy, with a high mortality rate (Mizrahi et al., 2020). Because of its early subclinical symptoms, the vast majority of PAAD patients were diagnosed in the middle to late stages, even with distant metastasis. Worse still, the level of acceptance of surgical treatment is relatively low at 20% (Singhi et al., 2019). Improving the prognosis of PAAD remains a challenge, with less than 5% of patients surviving for 5 years (Hidalgo, 2010; Hidalgo et al., 2015). In recent years, neoadjuvant therapy, targeted therapy, and immunotherapy have gained widespread application and yielded good clinical outcomes (Neoptolemos et al., 2018; Tempero, 2019).

Ferroptosis id a new type of cell death regulated by multiple metabolic pathways, lipid peroxide accumulation and iron dependence and, thus, may be involved in the mechanism of PAAD progression (Gupta et al., 2017; Wei et al., 2019; Badgley et al., 2020; Zhou et al., 2020). Emerging evidence suggests that ferroptosis provides a link between the pathophysiological disease mechanism and the altered human health status through its involvement in metabolic and redox reactions and negatively affects (Zheng and Conrad, 2020). It is promoted by BRCA1-associated protein 1 (BAP1) through SLC7A11 repression, and inhibited by glutathione-dependent phospholipid peroxidase 4, preventing lipid peroxide accumulation. Promoting ferroptosis circumvents therapy-resistant cancer cells (Hassannia et al., 2019). Therefore, a validated predictive model is needed to assess patient prognosis and guide patients to personalized targeted therapy.

Long non-coding RNAs (lncRNAs) are transcribed from genes but do not undergo translation into proteins (Bhan et al., 2017). They mediate the regulation of ferroptosis pathways; for instance, the cytoplasmic lncRNA P53RRA can promote ferroptosis through nuclear segregation of p53 (Mao et al., 2018). Furthermore, lncRNAs can interact with mRNA, thus modulating mRNA stability. Therefore, they are a crucial factor in regulating cholesterol metabolism processes in cancer.

Whether or not targeted therapies can have anti-cancer effects on patients needs to be further explored. A validated predictive model is required to accurately assess patient prognosis, account for individual differences, and guide individualized treatment to prolong survival.

The aim of the present study was to identify ferroptosis-related lncRNAs (FRLs) that have prognostic value in PAAD. We utilized The Cancer Genome Atlas (TCGA) dataset for lncRNA expression in PAAD to create a FRLs signature and elucidate its ability to predict overall survival (OS) in PAAD. Further, we summarize six previous reports of FRLs and briefly describe their potential mechanisms of action to provide a reference for further research.

## 2 Materials and Methods

### 2.1 Gene Expression and Clinical Data Access to Pancreatic Adenocarcinoma

Gene expression data and clinical information were downloaded from the TCGA-PAAD cohort FPKM file from the TCGA Information Portal (https://portal.gdc.cancer.gov/). After excluding 14 patients without RNA-seq or clinical information, 171 patients were included in this study. The 171 patients were divided into training cohort (*n* = 86) and testing cohort (*n* = 85). The training cohort were used for risk score model building, and the testing cohort and entire cohort were used for validation of the risk score model to test the robustness of the risk model.

TCGA data is open to the community and our research is based on the TCGA Data Access Policy and Publication Guidelines.

### 2.2 Screening for Ferroptosis-Related lncRNAs

The ferroptosis-related mRNAs were obtained from the FerrDb database (http://www.zhounan.org/ferrdb), which comprised a total of 239 genes. Then, Pearson’s correlation analysis was performed for FRLs identified using the “limma” package. LncRNAs with R2 > .6 and *p* < .001 were considered to be FRLs.

### 2.3 Construction of a Prognostic Ferroptosis-Related lncRNAs Signature

First, the training cohort was used to construct a prognostic FRLs signature. Co-expressed FRLs were tested using the univariate Cox analysis to determine the prognostic value. Subsequently, based on the candidate lncRNAs with *p* < .01 in the univariate Cox screen analysis, a least absolute shrinkage and selection operator (LASSO) regression model was constructed by “glmnet” package. Morever, the resulting lncRNAs were introduced into a multivariate Cox model to obtain hazard ratios (HR) and regression coefficients for each lncRNA used in the construction of the final prognostic FRLs signature using “survival” package. Risk scores were calculated using the following equation. The risk score was calculated using the following formula:

$$\begin{array}{l} \mathrm{Risk}\;\mathrm{score}=Exp_{\mathrm{lncRNA}1}\times \mathrm{Coe}f_{\mathrm{lncRNA}1}+Exp_{\mathrm{lncRNA}2}\times \mathrm{Coe}f_{\mathrm{lncRNA}2}\\ +\ldots +Exp_{\mathrm{lncRNAi}}\times \mathrm{Coe}f_{\mathrm{lncRNAi}} \end{array}$$

where $Exp_{lncRNAi}$ is the expression of the *i*th selected lncRNA, and ${\text{Coef}}_{lncRNAi}$ is its regression coefficient.

Subsequently, patients were divided into high- and low-risk groups based on the median risk score. The “survival” package was used to perform the Kaplan Meier (KM) survival and receiver operating characteristic (ROC) curve analysis of the performance of prognostic factors in terms of overall survival (OS). The testing cohort and the entire cohort were applied to validate the prognosis signature constructed based on the training cohort.

### 2.4 Assessment of Prognostic Factors

The KM analysis was performed in high-risk and low-risk groups to elucidate differences between subgroups based on gender (male and female), age (<65 and ≥65 years), grade (G1, G2 and G3, G4), T stage (T1, T2 and T3, T4) and N stage (N0 and N1). Univariate and multivariate Cox regression analysis of clinical characteristics and risk scores were performed to assess whether or not the risk score was an independent prognostic factor as well as to identify clinical characteristics that were independent prognostic factors.

### 2.5 Clinical Value of a Prognostic Ferroptosis-Related lncRNAs Signature

In the classification of clinicopathological characteristics, the age, sex, grade, American Joint Committee on Cancer (AJCC) stage, T-stage, and N-stage were used to elucidate the independent risk factors associated with the prognosis. However, the M-stage was excluded from the study because of the high rate of missing data on the M-stage.

### 2.6. Construction of a Predictive Nomogram

A nomogram was used to predict the 12-, 18-, and 24-month survival. The stability of the model was assessed using the consistency index (C-index) and calibration curves.

### 2.7 Ferroptosis-Related LncRNA-mRNA Co-Expression Network Construction

LncRNA-mRNA co-expression pairs with *R* ^2^ > .4 and *p* < 0.05 were considered to be potential regulatory pathways. Subsequently, the co-expression network was visualized by Cytoscape software (version 3.8.2, http://www.cytoscape.org/).

### 2.8 Functional Analysis

To elucidate potential biological functions of FRLs, Gene Ontology (GO) (Gene Ontology Consortium, 2015) and Kyoto Encyclopedia of Genes and Genomes (KEGG) (Kanehisa and Goto, 2000) was performed on lncRNA-mRNAs. Then, significantly related biological functions and pathways, which may be potential pathways for ferroptosis-related lncRNA regulation, were listed.

### 2.9 Statistical Analysis

Conversion of gene names and merging of the patient gene expression data with the clinical data were performed using PERL (version 5.30.2, http://www.perl.org/). All statistical analyses were performed in the R software (version 4.1.0).

## 3 Results

### 3.1 Patient Data Sets

**FIGURE 1:**
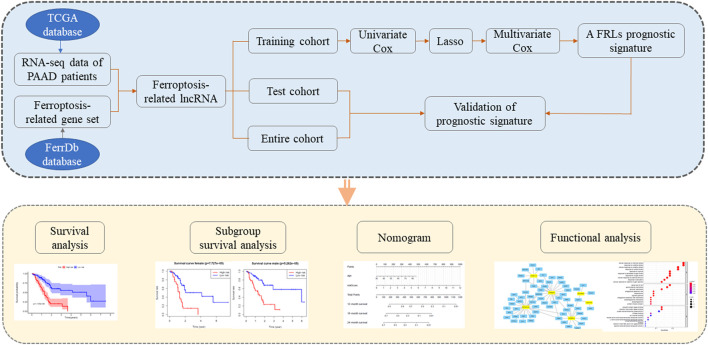
Flow chart of the study.

Data from the TCGA-PAAD cohort were downloaded and processed, as reported previously. Inclusion criteria were: 1) a histological diagnosis of pancreatic adenocarcinoma; 2) available data on gene expression and clinicopathological characteristics; and 3) complete survival information and a follow-up duration >30 days. When a patient had two or more samples then the first sample was selected. After applying inclusion and exclusion criteria, 171 patients were enrolled. Table 1 summarizes the basic patient profile. Then, the 171 PAAD patients were randomly divided into a training cohort (*n* = 86) and a testing cohort (*n* = 85) at a 1:1 ratio. The flow chart of the study is shown in Figure 1.

**TABLE 1:** Baseline table for TCGA-PAAD.

Clinical/Pathological features	Training cohort (*n* = 86)	Testing cohort (*n* = 85)	Entire cohort (*n* = 171)
Age			
<65	36	42	78
≥65	50	43	93
Gender			
Male	49	44	93
Female	37	41	78
Grade			
G1	15	13	28
G2	44	48	92
G3	25	22	47
G4	1	1	2
GX	1	1	2
Stage			
Stage I	1	0	1
Stage IA	3	2	5
Stage IB	8	5	13
Stage IIA	19	9	28
Stage IIB	52	62	114
Stage III	1	2	3
Stage IV	1	3	4
Unknown	1	2	3
T			
T1	4	3	7
T2	10	11	21
T3	70	68	138
T4	1	2	3
TX	0	1	1
Unknown	1	0	1
M			
M0	37	40	77
M1	1	3	4
MX	48	42	90
N			
N0	30	17	47
N1	50	65	115
N1b	3	1	4
NX	2	2	4
Unknown	1	0	1

### 3.2 Identification of Ferroptosis-Related lncRNAs in Pancreatic Adenocarcinoma

First, 13,830 lncRNAs were identified by analyzing gene expression data from TCGA-PAAD. Altogether 239 ferroptosis-related genes (Supplementary Table S1) were expressed in patients with PAAD. Finally, 435 FRLs were selected. (*R* ^2^ > 0.6 and *p* < .001).

### 3.3 Construction of a Prognostic Ferroptosis-Related lncRNAs Signature

**FIGURE 2:**
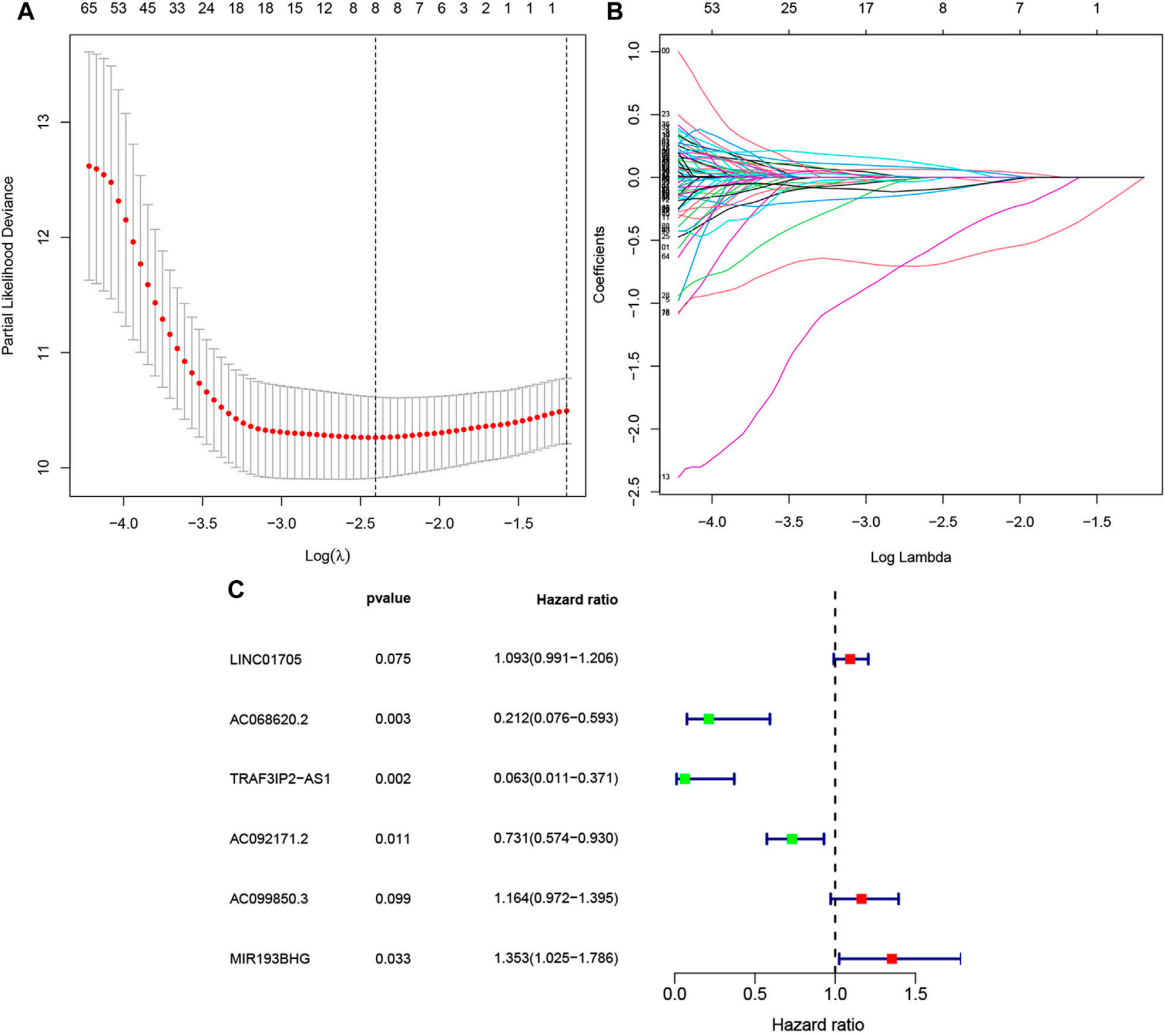
Selection of FRLs using LASSO and multivariate Cox regression. **(A)**Eight FRLs based LASSO cross validation plot. **(B)** LASSO coefficient of eight FRLs in PAAD. **(C)** Multivariate Cox regression showing six FRLs (LINC01705, AC068620.2, TRAF3IP2-AS1, AC092171.2, AC099850.3, and MIR193BHG) associated with OS in TCGA-PAAD.

The univariate Cox regression performed on 86 training PAAD samples with OS revealed 193 FRLs to be relevant with the PAAD prognosis (*p* < .01, Supplementary Table S2). Subsequently, LASSO regression of these genes, and eight FRLs were identified after LASSO regression (Figures 2A,B). Finally, multivariate Cox regression revealed six FRLs (LINC01705, AC068620.2, TRAF3IP2-AS1, AC092171.2, AC099850.3, and MIR193BHG, Figure 2C). A FRLs signature was created (Table 2) with the following risk score formula:

$$\begin{array}{l} \mathrm{Risk}\;\mathrm{score}=\left(\mathrm{ExpLINC}01705\times .0892\right)\\ -\left(\mathrm{ExpAC}068620.2*1.5521\right)\\ -\left(\mathrm{ExpTRAF}3\mathrm{IP}2-\mathrm{AS}1*2.7649\right)\\ -\left(\mathrm{ExpAC}092171.2*0.3138\right)\\ +\left(\mathrm{ExpAC}099850.3*0.1522\right)\\ +\left(\mathrm{ExpMIR}193\mathrm{BHG}*0.3023\right) \end{array}$$

**TABLE 2:** Multivariate Cox results for FRLs based on TCGA-PAAD.

Id	Coef	HR	HR.95L	HR.95H	*p*-value	Risk
LINC01705	.0892	1.0933	.9911	1.2060	.0748	High
AC068620.2	−1.5521	.2118	.0756	.5932	.0031	Low
TRAF3IP2-AS1	−2.7649	.0630	.0107	.3707	.0022	Low
AC092171.2	−.3138	.7307	.5742	.9298	.0107	Low
AC099850.3	.1522	1.1644	.9719	1.3951	.0988	High
MIR193BHG	.3023	1.3529	1.0246	1.7863	.0330	High

Table 3 summarizes previous reports on the six FRLs. To the best of our knowledge, two lncRNAs (AC068620.2 and AC092171.2) have not been previously reported.

**TABLE 3:** Summary statistics of available studies on FRLs.

Gene symbol	Evidence types	References
LINC01705	Cell culture; Mouse models; Clinical samples	Xie et al. (2018), Du et al. (2020), Lei Yang et al. (2021)
TRAF3IP2-AS1	Cell culture; Mouse models; Clinical samples; review; Bioinformatics Analysis	Zou et al. (2014), Bannon et al. (2015), Fabbri and Serretti, (2016), Zan and Li, (2019), He et al. (2021), Shuai Yang et al. (2021)
AC099850.3	Bioinformatics Analysis	Jia et al. (2020), Hao Wu et al. (2020), Jiang et al. (2021), Wu et al. (2021), Xu et al. (2021), Zhang et al. (2021), Zheng et al. (2021a), Junliang Zhou et al. (2021)
MIR193BHG	Cell culture; Bioinformatics Analysis; Clinical samples	Chan Meng et al. (2020), Xue Wu et al. (2020), Zhang et al. (2020), Zheng et al. (2021b), Qiwei Zhou et al. (2021)

### 3.4 Assessment of a Prognostic Ferroptosis-Related lncRNAs Signature

**FIGURE 3:**
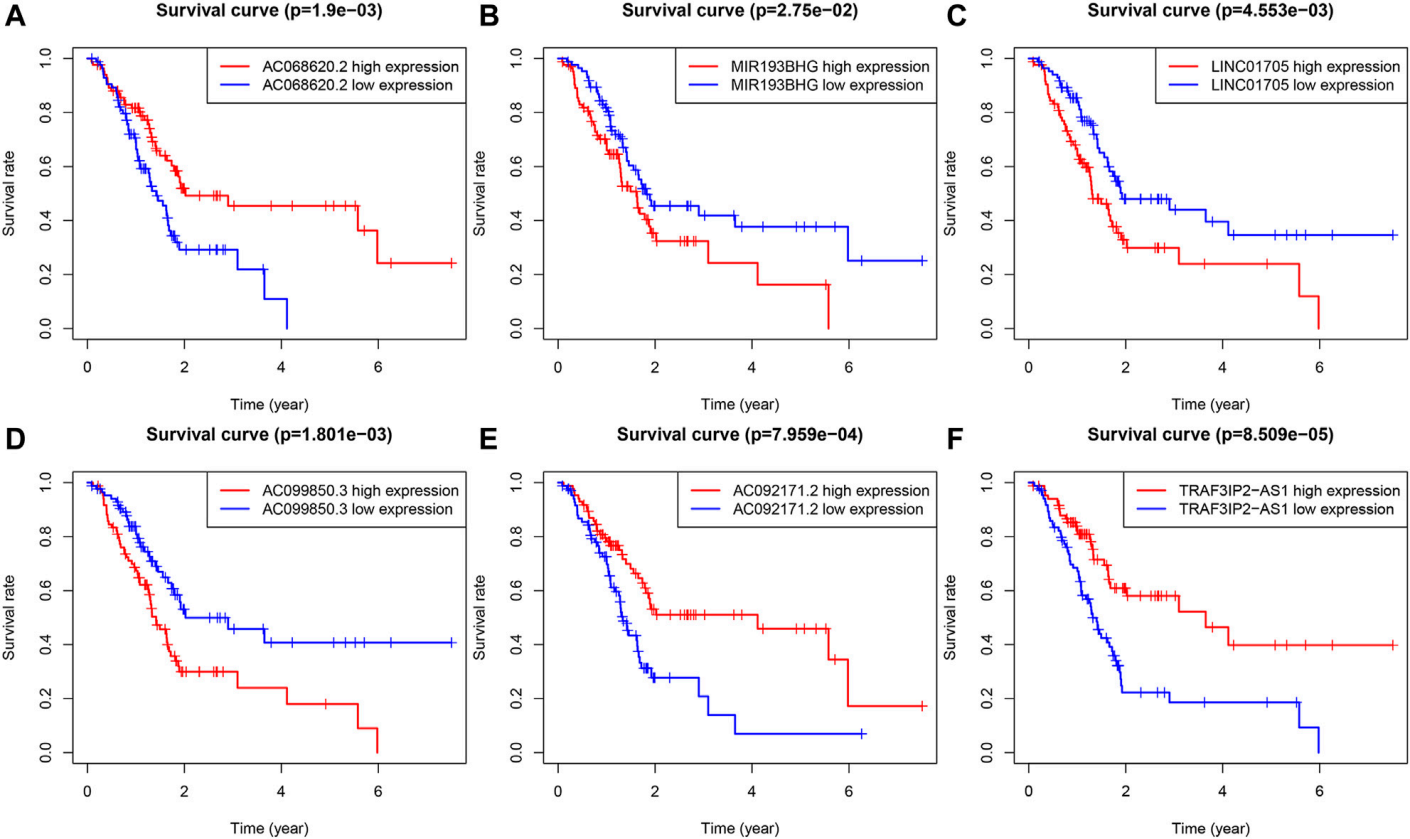
KM survival curves for the six prognostic FRLs. Three FRLs (LINC01705, AC099850.3 and MIR193BHG) were independent unfavorable factors and three lncRNAs (AC068620.2, TRAF3IP2-AS1, and AC092171.2) were independent favorable factors for PAAD.

**FIGURE 4:**
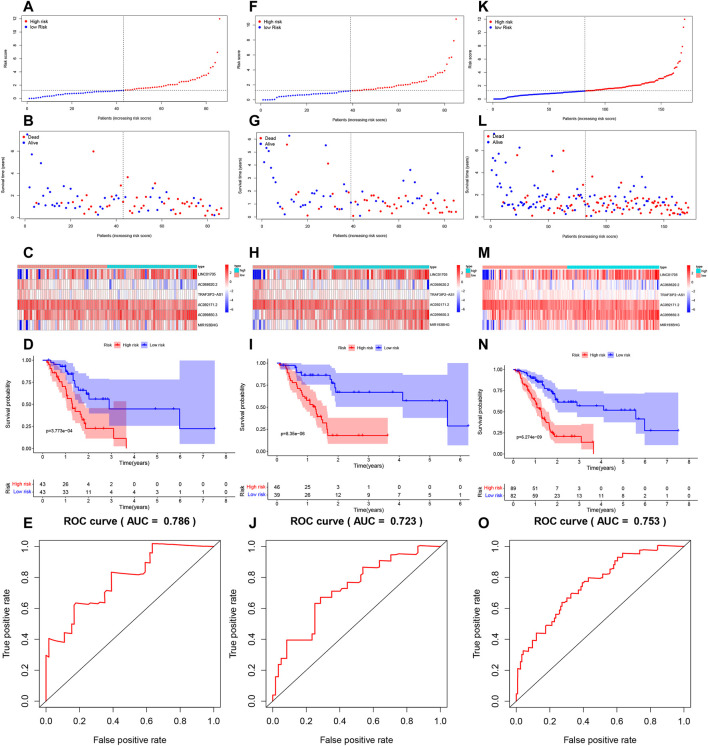
Risk score model development and validation. **(A)** Trianing cohort risk score distribution of a patient with PAAD based on FRLs. **(B)** Trianing cohort scatter plots showing the association between the OS and the risk score in PAAD patients according to prognostic features of FRLs. **(C)** Trianing cohort heatmap showing three unfavorable genes (LINC01705, AC099850.3, and MIR193BHG) with high expression in high-risk patients, contray to the expression of the three favorable genes (AC068620.2, TRAF3IP2-AS1, and AC092171.2). **(D)** Trianing cohort KM survival curve analysis. **(E)** Testing cohort area under the ROC curve based on FRLs-based prognostic features at 12 months. **(F)** Testing cohort risk score distribution of a patient with PAAD based on FRLs. **(G)** Testing cohort scatter plots showing the association between the OS and the risk score in PAAD patients according to prognostic features of FRLs. **(H)** Testing cohort heatmap showing three unfavorable genes (LINC01705, AC099850.3, and MIR193BHG) with high expression in high-risk patients, contray to the expression of the three favorable genes (AC068620.2, TRAF3IP2-AS1, and AC092171.2). **(I)** Testing cohort KM survival curve analysis. **(J)** Testing cohort area under the ROC curve based on FRLs-based prognostic features at 12 months. **(K)** Entire cohort risk score distribution of a patient with PAAD based on FRLs. **(L)** Entire cohort scatter plots showing the association between the OS and the risk score in PAAD patients according to prognostic features of FRLs. **(M)** Entire cohort heatmap showing three unfavorable genes (LINC01705, AC099850.3, and MIR193BHG) with high expression in high-risk patients, contray to the expression of the three favorable genes (AC068620.2, TRAF3IP2-AS1, and AC092171.2). **(N)** Entire cohort KM survival curve analysis. **(O)** Entire cohort area under the ROC curve based on FRLs-based prognostic features at 12 months.

LINC01705, AC099850.3, and MIR193BHG were risk factors for PAAD, while three lncRNAs (AC068620.2, TRAF3IP2-AS1, and AC092171.2) were protective factors for PAAD (Figures 3A–F). The risk score formula applied to gene expression data of 86 training patients revealed a median risk score of 1.231. Accordingly, all cases could be classified as low-risk (*n* = 43) or high-risk (*n* = 43) (Figure 4A). The increased risk score was accompanied by gradually decreasing survival time and increased mortality (Figure 4B). Finally, the expression level of OS-related FRLs was presented in the form of heatmaps (Figure 4C). Regrettably, none of the patients in the high-risk group survived longer than 4 years, whereas many of those in the low-risk group survived longer than 7 years. After log-rank test, there was a significant difference in OS between the two groups (*p* = 1.135e-08, Figure 4D). Time-dependent ROC curves showed prediction of lncRNA biomarkers in the prognosis, with AUC of 0.786, respectively (Figure 4E). We then validated the a prognostic FRLs signature on the testing cohort and the entire cohort (Figures 4F–O). Survival analysis showed, significant differences in survival time between both the testing cohort and the entire cohort of patients, with significantly higher OS in the low-risk group than in the high-risk group. In addition, the AUCs for the testing cohort and the entire cohort were .723, .753, respectively, showing that the six FRLs prognostic signature has good robustness.

### 3.5Clinical Value of a Prognostic Ferroptosis-Related lncRNAs Signature

**FIGURE 5:**
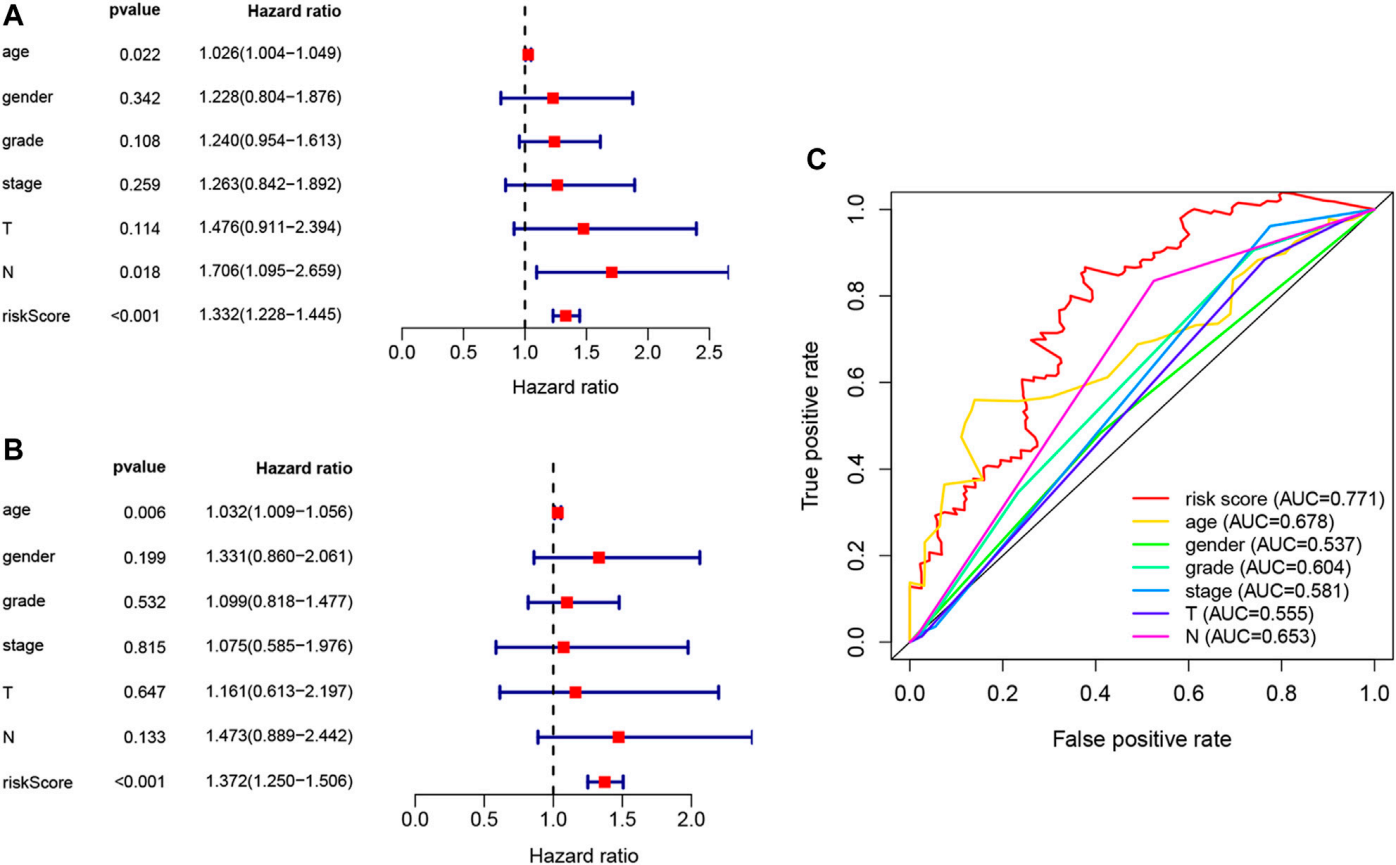
Estimated prognostic accuracy of FRLs in patients with PAAD. **(A)** Univariate Cox regression showing that the age, N-stage, and risk score were associated with OS (*p* < .05). **(B)** Multivariate Cox regression showing that the age and the risk score (*p* < .01) were independent prognostic indicators of OS in patients with PAAD. **(C)** ROC curve showing that the risk score has the highest prognostic accuracy.

**FIGURE 6:**
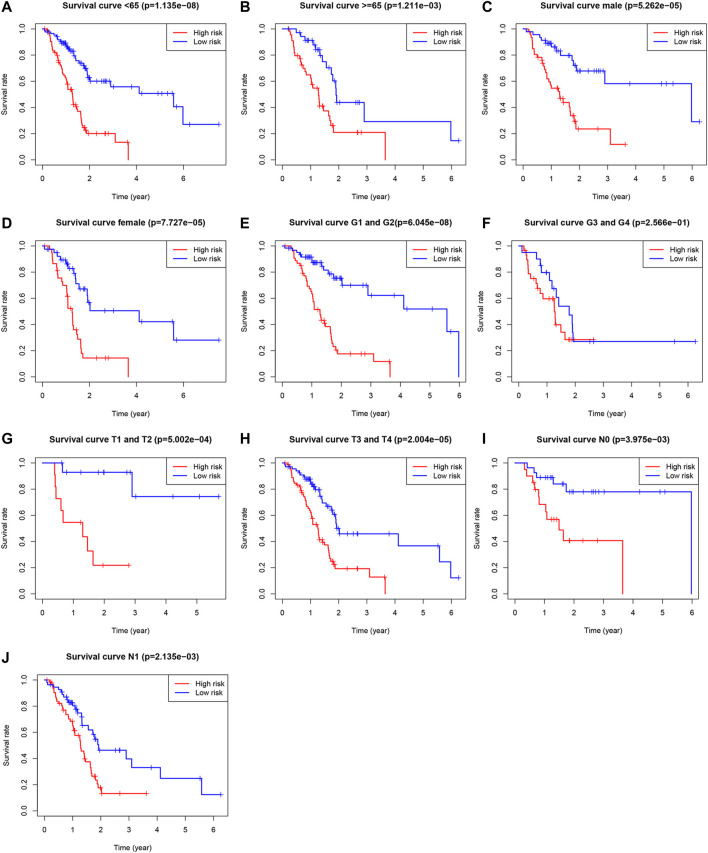
Survival rates of PAAD patients with high- and low-risk patients with PAAD in the subgroups based on clinicopathological characteristics. **(A)** Subgroup of age <65 years. **(B)** Subgroup of age ≥65 years. **(C)** Male subgroup. **(D)** Female subgroup. **(E)** G1 and G2 subgroups. **(F)** G3 and G4 subgroups. **(G)** T1 and T2 subgroups. **(H)** T3 and T4 subgroups. **(I)** N0 subgroup. **(J)** N1 subgroup.

Univariate Cox, regression revealed the risk score to be a risk factor significantly associated with the prognosis of patients with PAAD (95% confidence interval (CI): 1.228–1.445, *p* < .001), with a higher risk score suggesting a worse prognosis. In addition, the N-stage (95% CI: 1.095–2.659, *p* = .018) and patient age (95% CI: 1.004–1.049, *p* = .022) were also closely associated with the prognosis. (Figure 5A). After controlling for clinical characteristics, risk score remained an independent indicator (hazard ratio = 1.372, 95% CI: 1.250–1.506, *p* < .001, Figure 5B, Table 4). However, the N-stage was not significantly associated with the prognosis in multivariate Cox regression. Moreover, higher accuracy of the risk score in predicting patients compared to other clinicopathological features was also confirmed in the ROC analysis. The prognostic value of FRLs (.771) was higher than that of age (.678), sex (.537), grade (.604), the AJCC stage (.581), the T-stage (.555), and the N-stage (.653; Figure 5C). Similarly, across all age, sex, T-stage, grade, and AJCC stage subgroups, patients with PAAD showed a lower OS rate in the high-risk group than in the low-risk group (Figure 6). All data suggested that the prognostic FRLs signature is significantly associated with the prognosis of patients with PAAD.

**TABLE 4:** Multivariate Cox regression analysis of clinical characteristics and the risk score for PAAD.

Id	HR	HR.95L	HR.95H	*p*-value
Age	1.032	1.009	1.056	.006
Gender	1.328	.856	2.060	.206
Grade	1.099	.818	1.476	.532
Stage	1.076	.586	1.978	.813
T	1.155	.607	2.199	.661
M	.987	.792	1.229	.903
N	1.477	.890	2.450	.132
RiskScore	1.372	1.250	1.506	.000

### 3.6 Construction and Assessment of Nomogram

**FIGURE 7:**
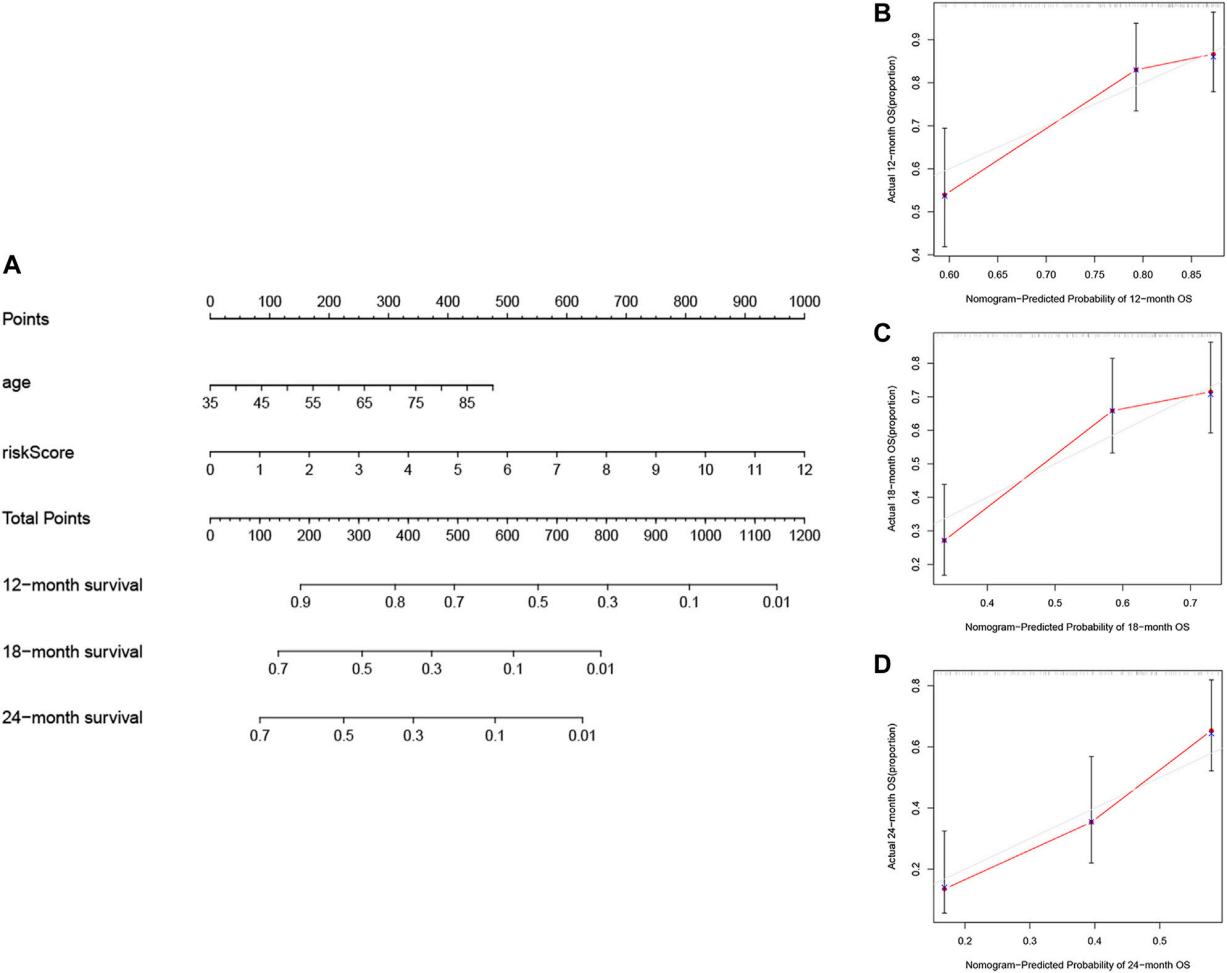
Construction and validation of the nomogram. **(A)** Prognostic nomogram based on the risk score using the prognostic FRLs signature and the age to predict 12-, 18-, and 24-month survival in patients with PAAD. Calibration curves corrected for deviations in agreement between the predicted and observed survival rates at **(B)** 12, **(C)** 18 and **(D)** 24-months.

The nomogram, a common prognostic visualization tool in oncology, allows for the quantification of patient survival based on the inclusion of an index score. Based on multivariate Cox regression, the indicators, age and risk score, were included in the construction of the nomogram. Total scores were obtained by adding the individual indicator scores for age and risk score and predicting the probability of survival at 12, 18, and 24 months (Figure 7A). The nomogram had good stability at a consistency index of .697. Furthermore, calibration curves showed that predicted survival times at 12, 18, and 24 months were consistent compared to the reference line (Figures 7B–D). Furthermore, Table 5 lists other studies in which lncRNA predicted the prognosis of patients with PAAD.

**TABLE 5:** Comparative studies on available lncRNA signatures in PAAD.

Feature	Database	Gene list	OS	AUC	Refer
All lncRNA	TCGA	AC009014.3, RP11-48O20.4, UCA1	OS	.689	Li et al. (2020)
	ICGC				
Immune-related lncRNA	TCGA	MANCR, LINC01655, UCA1, LINC01940, FIRRE, LINC01776, LINC01436, LINC00242	OS	.74	Qi et al. (2021)
M6A-related lncRNA	TCGA	AC099850.3, UCA1, AP005233.2, AL513165.1, PTOV1-AS2	OS	.682	Yuan et al. (2021)
	ICGC				
	GTEx				
All lncRNA	GEO	lA2M-AS1, DLEU2, LINC01133, LINC00675, MIR155HG, SLC25A25-AS1, LINC01857, LOC642852(LINC00205), ITGB2-AS1, TSPOAP1-DACAS1, PSMB2-AS1	—	—	Giulietti et al. (2018)
All lncRNA	TCGA	AL137789.1, MIR600HG, AC079015.1	OS	.742	Wu et al. (2018)
All lncRNA	ICGC	AC009014.3, RP11-48O20.4, UCA1	—	—	Li et al. (2020)
CeRNA	GEO	SCAMP1, HCP5, MAL2, LINC00511	—	—	Wang et al. (2019)

### 3.7 Construction of the Prognostic Ferroptosis-Related lncRNAs Associated LncRNA-mRNA Co-Expression Network and the Functional Enrichment Analysis

**FIGURE 8:**
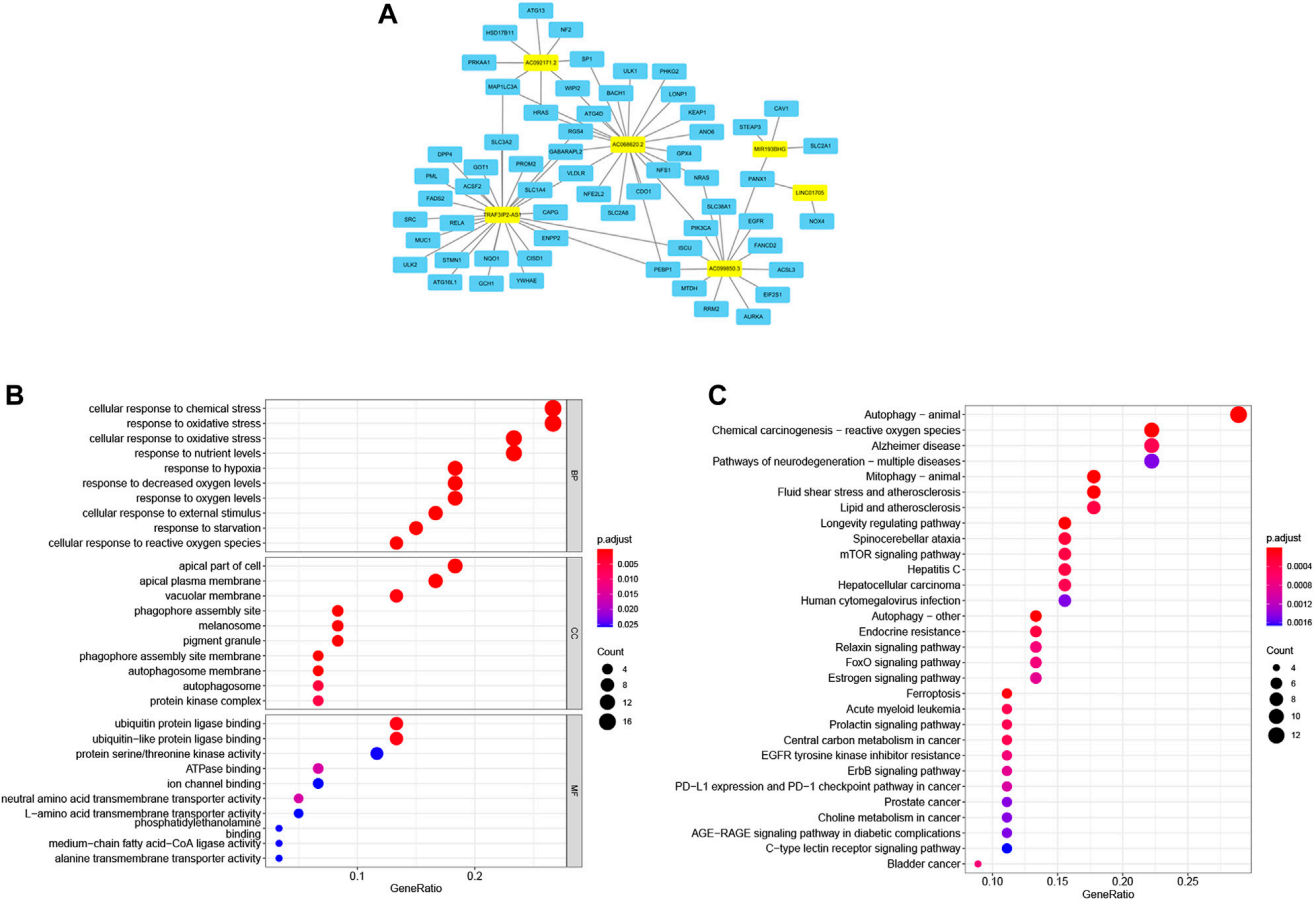
Ferroptosis-related lncRNA-mRNA co-expression network and functional enrichment. **(A)** LncRNA-mRNA network showing 76 lncRNA-mRNA co-expression pairs formed between six FRLs and 61 mRNAs. The yellow rectangles denote FRLs, and the blue rectangles denote mRNAs. **(B)** GO analysis showing enriched biological function of these mRNAs co-expressed with six FRLs. **(C)** KEGG pathway analysis showing enriched signaling pathways.

The co-expression analysis of FRLs revealed a total of 61 mRNAs involved in formation of the co-expression network of 76 lncRNA-mRNA targets with *R* ^2^ > .4 and *p* < .05 (Figure 8A; Supplementary Table S3). The GO analysis showed that it was involved in 476 biological processes, such as cellular responses to chemical stress and responses to oxidative stress, constituted 25 cellular components, including the phagosome assembly site and the apical portion of cells, and mediated 35 molecular functions, such as ubiquitin protein ligase binding and ubiquitin-like protein ligase binding (Figure 8B; Table 6). KEGG showed ferroptosis to be a significantly enriched pathway (Figure 8C; Table 7).

**TABLE 6:** Significantly enriched GO terms of co-expression mRNA with PAAD with an adjusted *p* < .05

Category	Term	Count	*p*-value	Adj *p*-value
BP	GO:0062197∼ cellular response to chemical stress	16	<.001	<.001
BP	GO:0006979∼ response to oxidative stress	16	<.001	<.001
BP	GO:0034599∼ cellular response to oxidative stress	14	<.001	<.001
BP	GO:0031667∼ response to nutrient levels	14	<.001	<.001
BP	GO:0001666∼ response to hypoxia	11	<.001	<.001
BP	GO:0042594∼ response to starvation	9	<.001	<.001
BP	GO:0036293∼ response to decreased oxygen levels	11	<.001	<.001
BP	GO:0070482∼ response to oxygen levels	11	<.001	<.001
BP	GO:0034614∼ cellular response to reactive oxygen species	8	<.001	<.001
BP	GO:0071496∼ cellular response to external stimulus	10	<.001	<.001
CC	GO:0000407∼phagophore assembly site	5	<.001	<.001
CC	GO:0045177∼apical part of cell	11	<.001	<.001
CC	GO:0016324∼apical plasma membrane	10	<.001	<.001
CC	GO:0034045∼phagophore assembly site membrane	4	<.001	<.001
CC	GO:0000421∼autophagosome membrane	4	<.001	<.001
CC	GO:0042470∼melanosome	5	<.001	<.001
CC	GO:0048770∼pigment granule	5	<.001	<.001
CC	GO:0005774∼vacuolar membrane	8	<.001	.001
CC	GO:0005776∼autophagosome	4	<.001	.005
CC	GO:1902911∼protein kinase complex	4	<.001	.006
MF	GO:0031625∼ubiquitin protein ligase binding	8	<.001	.001
MF	GO:0044389∼ubiquitin-like protein ligase binding	8	<.001	.001
MF	GO:0015175∼neutral amino acid transmembrane transporter activity	3	<.001	.016
MF	GO:0051117∼ATPase binding	4	<.001	.016
MF	GO:0004674∼protein serine/threonine kinase activity	7	.001	.026
MF	GO:0008429∼phosphatidylethanolamine binding	2	.001	.026
MF	GO:0031956∼medium-chain fatty acid-CoA ligase activity	2	.001	.026
MF	GO:0022858∼alanine transmembrane transporter activity	2	.001	.026
MF	GO:0044325∼ion channel binding	4	.001	.026
MF	GO:0015179∼L-amino acid transmembrane transporter activity	3	.001	.026

**TABLE 7:** Signifcantly enriched KEGG terms of co-expression mRNA with PAAD with an adjusted *p* < .05

KEGG term	Count	*p*-value	Adj *p*-value
hsa04140∼Autophagy—animal	13	<.001	<.001
hsa04137∼Mitophagy—animal	8	<.001	<.001
hsa04136∼Autophagy—other	6	<.001	<.001
hsa05208∼Chemical carcinogenesis—reactive oxygen species	10	<.001	<.001
hsa04211∼Longevity regulating pathway	7	<.001	<.001
hsa05418∼Fluid shear stress and atherosclerosis	8	<.001	<.001
hsa04216∼Ferroptosis	5	<.001	<.001
hsa05017∼Spinocerebellar ataxia	7	<.001	<.001
hsa01522∼Endocrine resistance	6	<.001	<.001
hsa05417∼Lipid and atherosclerosis	8	<.001	<.001
hsa04150∼mTOR signaling pathway	7	<.001	<.001
hsa05160∼Hepatitis C	7	<.001	<.001
hsa05221∼Acute myeloid leukemia	5	<.001	<.001
hsa05163∼Human cytomegalovirus infection	7	<.001	.001
hsa04933∼AGE-RAGE signaling pathway in diabetic complications	5	<.001	.001
hsa05022∼Pathways of neurodegeneration—multiple diseases	10	<.001	.001
hsa04625∼C-type lectin receptor signaling pathway	5	<.001	.002

## 4 Discussion

PAAD is increasing in incidence every year and is highly malignant. It has a low early clinical detection rate and a 5-year survival rate <5%. Previous studies have elucidated prognostic factors associated with PAAD, such as the tumor grade, stage, size, and number. PAAD involves complex biological processes, some of which are closely related to its prognosis, such as autophagy (Yue et al., 2020), immunity, and ferroptosis. LncRNAs have been identified as key regulators of biological processes that could be used as potential prognostic biomarkers and might provide insights into clinically targeted therapies. In our study, the combination analysis of Cox and LASSO regression was applied to establish a ferroptosis-related lncRNA signature. The signature showed good predictive performance, with patients in the low-risk group having higher OS than those in the high-risk group.

Six prognosis-associated lncRNAs finally obtained in the risk model were LINC01705, AC068620.2, TRAF3IP2-AS1, AC092171.2, AC099850.3, and MIR193BHG. These FRLs could be prognostic marker molecules, potential markers for PAAD, and potential therapeutic targets.

Among the six ferroptosis-related gene risk models, AC084018.1 and AC092171.2 have not been reported to date. Regulations of AC084018.1 and AC092171.2 should be investigated in future studies. The biological mechanism of the other FRLs has been previously reported. LINC01705 positively regulates the translocation promoter region nuclear basket protein by competitively binding to miR-186-5p, thereby promoting the aggressiveness of breast cancer cells (Du et al., 2020). Moreover, miR-223-5p-LINC01705 is involved in pulmonary metastasis of osteosarcoma as a microRNA–lncRNA target pair (Lei Yang et al., 2021). In addition, LINC01705 has high expression during the pathogenesis of proliferative vitreoretinopathy (Xie et al., 2018). Shuai Yang et al. (2021) investigated the inhibitory effect of the tumor growth suppressor TRAF3IP2-AS1 on the progression of NONO-TFE3-translocated renal carcinoma and found that the overexpression of TRaf3IP2-AS1 could stimulate N6-methyladenosine of PARP1 mRNA and downregulate PTEN, further inhibiting the progression of renal carcinoma. He et al. (2021) showed that TRAF3IP2-AS1 may be an attractive therapeutic target for IL-17-related autoimmune diseases, such as psoriasis and multiple sclerosis. Bannon et al. (2015) analyzed clinical samples and showed that TRAF3IP2-AS1 exhibits high expression in cocaine abusers, positively correlating with the opposite chain protein encoding transcript TRAF3IP2. In the study by Zou et al. (2014), TRAF3IP2-AS1 expression was altered in gastric cancer cells after ^125^I irradiation, providing a target for future drug development. AC099850.3 has been repeatedly extracted as a prognosis-related gene in the bioinformatics analysis of cancer, including squamous cell carcinoma of the tongue, hepatocellular carcinoma, and non-small cell lung cancer (Hao Wu et al., 2020; Jia et al., 2020; Zheng et al., 2021a; Junliang Zhou et al., 2021; Jiang et al., 2021; Wu et al., 2021; Xu et al., 2021; Zhang et al., 2021). The MIR193BHG motif can fine-tune cellular sterol/steroid biosynthesis by producing lincNORS to repress the expression of multiple pathway components (Xue Wu et al., 2020). MIR193BHG was found to be significantly associated with the prognosis in both autophagy-related lncRNA in ovarian cancer and ferroptosis-related lncRNA in lung adenocarcinoma (Chan Meng et al., 2020; Zheng et al., 2021b). In the analysis of competing endogenous RNAs in lung and renal cell carcinomas, MIR193BHG-miR-140-3p may be a promising upstream regulatory pathway for GPRIN1 (Qiwei Zhou et al., 2021). Furthermore, MIR193BHG has diagnostic value for placental tissue in patients with early-onset pre-eclampsia (Zhang et al., 2020).

KEGG and GO enrichment results indicated that these screened lncRNAs were related to regulation of reactive oxygen species (ROS), which can cause ptosis. The effect of ROS on ferroptosis in pancreatic cancer is not fully understood. However, many regulators of ROS related to ferroptosis, such as irisin, deerskin, QD394, and QD394-me, have been found. ROS metabolism, iron metabolism, and the cysteine/glutamate reverse transport system (SYSTEM Xc-) play important roles in the regulatory process (Hu et al., 2020; Song et al., 2020; Yang and Leung, 2020). In terms of environment, many studies have shown that arsenic may stimulate ferroptosis in cancer cells through oxidative stress. Our enrichment analysis revealed that the co-expressed mRNA was significantly enriched in the autophagy pathway (Pan Meng et al., 2020; Wei et al., 2020). To date, the genetic link between autophagy and ferroptosis remains unclear. Autophagy has been shown to promote ferroptosis by degrading ferritin in cancer cells (Hou et al., 2016). Therefore, identifying lncRNAs associated with iron sagging in pancreatic cancer may provide guidance for the search for regulatory factors.

Ours was the first study to combine patient prognostic analysis with FRLs to preliminarily explore the molecular mechanism of ferroptosis affecting the prognosis of patients with PAAD. Six FRLs signatures were established to further supplement the traditional clinical prognostic factors, guiding the search for therapeutic targets and the selection of prognostic decisions for pancreatic cancer. In addition, we provided a columnar line graph correlating FRLs with clinical factors to predict OS in PAAD in a validated and quantitative way.

## 5 Conclusion

We created a ferroptosis-related lncRNAs signature that can be used as a new biomarker to predict PAAD progression. This signature could help provide insights into the correlation between ferroptosis and tumorigenesis for clinical ferroptosis-related targeted therapies.

## Data Availability

Publicly available datasets were analyzed in this study. This data can be found here: https://portal.gdc.cancer.gov/.

## Abbreviations

AUC, area under the receiver operating characteristic curve; FRLs, ferroptosis-related lncRNAs; GO, Gene Ontology; KEGG, Kyoto Encyclopedia of Genes and Genomes; LASSO, least absolute shrinkage and selection operator; OS, overall survival; PAAD, pancreatic adenocarcinoma; ROC, receiver operating characteristic; TCGA, The Cancer Genome Atlas.
