# Supplementary material for: Prognostication of Pancreatic Cancer Using The Cancer Genome Atlas Based Ferroptosis-Related Long Non-Coding RNAs
Source: Front Genet. 2022 Feb 14;13:838021. doi: 10.3389/fgene.2022.838021 (PMC8883032; doi:10.3389/fgene.2022.838021)
Supplement: Supplementary file 1 [file DataSheet1.docx]

**Supplementary Material**

Supplementary Table 1.Ferroptosis-related genes obtained from FerrDb database.

| Gene Code | Gene Symbol | Gene Code | Gene Symbol | Gene Code | Gene Symbol |
| --- | --- | --- | --- | --- | --- |
| 1 | RPL8 | 81 | TGFBR1 | 161 | SLC40A1 |
| 2 | IREB2 | 82 | EPAS1 | 162 | GPX4 |
| 3 | ATP5MC3 | 83 | HILPDA | 163 | HAMP |
| 4 | CS | 84 | HIF1A | 164 | HSPB1 |
| 5 | EMC2 | 85 | IFNG | 165 | NFE2L2 |
| 6 | ACSF2 | 86 | ANO6 | 166 | STEAP3 |
| 7 | NOX1 | 87 | LPIN1 | 167 | DRD5 |
| 8 | CYBB | 88 | HMGB1 | 168 | DRD4 |
| 9 | NOX3 | 89 | TNFAIP3 | 169 | MAP3K5 |
| 10 | NOX4 | 90 | TLR4 | 170 | SLC2A1 |
| 11 | NOX5 | 91 | ATF3 | 171 | SLC2A3 |
| 12 | DUOX1 | 92 | ATM | 172 | SLC2A6 |
| 13 | DUOX2 | 93 | YY1AP1 | 173 | SLC2A8 |
| 14 | G6PD | 94 | EGLN2 | 174 | SLC2A12 |
| 15 | PGD | 95 | MIOX | 175 | SLC2A14 |
| 16 | VDAC2 | 96 | TAZ | 176 | EIF2AK4 |
| 17 | PIK3CA | 97 | MTDH | 177 | TFAP2C |
| 18 | FLT3 | 98 | IDH1 | 178 | SP1 |
| 19 | SCP2 | 99 | SIRT1 | 179 | HBA1 |
| 20 | TP53 | 100 | FBXW7 | 180 | NNMT |
| 21 | ACSL4 | 101 | PANX1 | 181 | PLIN4 |
| 22 | LPCAT3 | 102 | DNAJB6 | 182 | HIC1 |
| 23 | NRAS | 103 | BACH1 | 183 | STMN1 |
| 24 | KRAS | 104 | LONP1 | 184 | RRM2 |
| 25 | HRAS | 105 | PTGS2 | 185 | CAPG |
| 26 | TF | 106 | DUSP1 | 186 | HNF4A |
| 27 | TFRC | 107 | NOS2 | 187 | NGB |
| 28 | TFR2 | 108 | NCF2 | 188 | YWHAE |
| 29 | SLC38A1 | 109 | MT3 | 189 | GABPB1 |
| 30 | SLC1A5 | 110 | UBC | 190 | AURKA |
| 31 | GLS2 | 111 | ALB | 191 | RIPK1 |
| 32 | GOT1 | 112 | TXNRD1 | 192 | PRDX1 |
| 33 | ALOX5 | 113 | SRXN1 | 193 | AKR1C1 |
| 34 | KEAP1 | 114 | GPX2 | 194 | AKR1C2 |
| 35 | HMOX1 | 115 | BNIP3 | 195 | AKR1C3 |
| 36 | ATG5 | 116 | OXSR1 | 196 | RB1 |
| 37 | ATG7 | 117 | SELENOS | 197 | HSF1 |
| 38 | NCOA4 | 118 | ANGPTL7 | 198 | GCLC |
| 39 | ALOX12 | 119 | SLC7A11 | 199 | SQSTM1 |
| 40 | ALOX12B | 120 | DDIT4 | 200 | NQO1 |
| 41 | ALOX15 | 121 | ASNS | 201 | MUC1 |
| 42 | ALOX15B | 122 | TSC22D3 | 202 | MT1G |
| 43 | ALOXE3 | 123 | DDIT3 | 203 | CISD1 |
| 44 | PHKG2 | 124 | JDP2 | 204 | FANCD2 |
| 45 | ACO1 | 125 | SESN2 | 205 | FTMT |
| 46 | ULK1 | 126 | SLC1A4 | 206 | HSPA5 |
| 47 | ATG3 | 127 | PCK2 | 207 | HELLS |
| 48 | ATG4D | 128 | TXNIP | 208 | SCD |
| 49 | BECN1 | 129 | VLDLR | 209 | FADS2 |
| 50 | MAP1LC3A | 130 | GPT2 | 210 | SRC |
| 51 | GABARAPL2 | 131 | PSAT1 | 211 | STAT3 |
| 52 | GABARAPL1 | 132 | LURAP1L | 212 | PML |
| 53 | ATG16L1 | 133 | SLC7A5 | 213 | MTOR |
| 54 | WIPI1 | 134 | HERPUD1 | 214 | NFS1 |
| 55 | WIPI2 | 135 | SLC3A2 | 215 | TP63 |
| 56 | SNX4 | 136 | CBS | 216 | CDKN1A |
| 57 | ATG13 | 137 | ATF4 | 217 | ENPP2 |
| 58 | ULK2 | 138 | ZNF419 | 218 | FH |
| 59 | SAT1 | 139 | KLHL24 | 219 | CISD2 |
| 60 | EGFR | 140 | TRIB3 | 220 | ISCU |
| 61 | MAPK3 | 141 | ZFP69B | 221 | ACSL3 |
| 62 | MAPK1 | 142 | ATP6V1G2 | 222 | OTUB1 |
| 63 | BID | 143 | VEGFA | 223 | CD44 |
| 64 | ZEB1 | 144 | GDF15 | 224 | BRD4 |
| 65 | DPP4 | 145 | TUBE1 | 225 | PRDX6 |
| 66 | CDKN2A | 146 | ARRDC3 | 226 | NF2 |
| 67 | PEBP1 | 147 | CEBPG | 227 | ARNTL |
| 68 | SOCS1 | 148 | RGS4 | 228 | JUN |
| 69 | CDO1 | 149 | BLOC1S5-TXNDC5 | 229 | CA9 |
| 70 | MYB | 150 | EIF2S1 | 230 | TMBIM4 |
| 71 | MAPK8 | 151 | IL6 | 231 | PLIN2 |
| 72 | MAPK9 | 152 | CXCL2 | 232 | AIFM2 |
| 73 | CHAC1 | 153 | RELA | 233 | LAMP2 |
| 74 | MAPK14 | 154 | HSD17B11 | 234 | ZFP36 |
| 75 | PRKAA2 | 155 | AGPAT3 | 235 | PROM2 |
| 76 | PRKAA1 | 156 | SETD1B | 236 | CHMP5 |
| 77 | ELAVL1 | 157 | FTL | 237 | CHMP6 |
| 78 | BAP1 | 158 | MAFG | 238 | CAV1 |
| 79 | ABCC1 | 159 | IL33 | 239 | GCH1 |
| 80 | ACVR1B | 160 | FTH1 |  |  |

Supplementary Table 2. Univariate Cox regression analysis of clinical characteristics and risk score for pancreatic cancer.

| Id | HR | HR.95L | HR.95H | *P*-value |
| --- | --- | --- | --- | --- |
| AC011352.3 | 1.614 | 1.151 | 2.263 | 0.005 |
| LINC01705 | 1.182 | 1.094 | 1.279 | 0.000 |
| LINC01943 | 1.905 | 1.222 | 2.969 | 0.004 |
| AC008608.2 | 0.721 | 0.573 | 0.908 | 0.005 |
| AC005785.1 | 0.319 | 0.166 | 0.612 | 0.001 |
| SNHG11 | 0.736 | 0.586 | 0.925 | 0.009 |
| OIP5-AS1 | 1.295 | 1.086 | 1.545 | 0.004 |
| ASB16-AS1 | 0.711 | 0.575 | 0.879 | 0.002 |
| LINC01023 | 0.827 | 0.730 | 0.935 | 0.003 |
| AC011462.4 | 0.688 | 0.529 | 0.895 | 0.005 |
| AC073046.1 | 1.923 | 1.283 | 2.883 | 0.002 |
| AC010531.6 | 0.598 | 0.432 | 0.827 | 0.002 |
| ARRDC1-AS1 | 0.775 | 0.665 | 0.904 | 0.001 |
| AC007292.1 | 0.427 | 0.240 | 0.760 | 0.004 |
| AC106820.3 | 0.465 | 0.266 | 0.814 | 0.007 |
| AC020765.2 | 0.384 | 0.217 | 0.679 | 0.001 |
| AL451085.2 | 0.446 | 0.263 | 0.757 | 0.003 |
| AC109322.1 | 0.522 | 0.328 | 0.829 | 0.006 |
| CAHM | 0.305 | 0.146 | 0.639 | 0.002 |
| AL121832.2 | 0.760 | 0.635 | 0.908 | 0.003 |
| AC139530.1 | 0.536 | 0.380 | 0.756 | <0.001 |
| YTHDF3-AS1 | 0.599 | 0.430 | 0.834 | 0.002 |
| AC040169.1 | 0.604 | 0.451 | 0.808 | 0.001 |
| H1FX-AS1 | 0.312 | 0.151 | 0.643 | 0.002 |
| AC138207.2 | 0.500 | 0.320 | 0.780 | 0.002 |
| AC034236.2 | 0.433 | 0.265 | 0.708 | 0.001 |
| AC068580.1 | 1.113 | 1.030 | 1.203 | 0.007 |
| AC124016.1 | 0.317 | 0.153 | 0.658 | 0.002 |
| AC245884.8 | 0.697 | 0.531 | 0.914 | 0.009 |
| SNHG10 | 0.636 | 0.483 | 0.837 | 0.001 |
| LINC00685 | 0.692 | 0.528 | 0.907 | 0.008 |
| AL139089.1 | 0.576 | 0.392 | 0.845 | 0.005 |
| AC008610.1 | 0.738 | 0.605 | 0.900 | 0.003 |
| AC092171.4 | 0.589 | 0.421 | 0.825 | 0.002 |
| AC132192.2 | 0.609 | 0.421 | 0.881 | 0.008 |
| AC006942.1 | 0.639 | 0.478 | 0.854 | 0.002 |
| AC108673.3 | 0.783 | 0.661 | 0.926 | 0.004 |
| LINC00641 | 0.549 | 0.367 | 0.820 | 0.003 |
| DICER1-AS1 | 0.601 | 0.436 | 0.829 | 0.002 |
| AC020558.2 | 0.567 | 0.374 | 0.859 | 0.007 |
| AC063948.1 | 0.467 | 0.294 | 0.742 | 0.001 |
| SNHG7 | 0.899 | 0.830 | 0.973 | 0.008 |
| AL109811.3 | 0.667 | 0.539 | 0.826 | 0.000 |
| AC005076.1 | 0.476 | 0.295 | 0.767 | 0.002 |
| AC084018.1 | 0.731 | 0.612 | 0.874 | 0.001 |
| AC005393.1 | 0.475 | 0.292 | 0.771 | 0.003 |
| ILF3-DT | 0.884 | 0.819 | 0.953 | 0.001 |
| GEMIN7-AS1 | 0.258 | 0.117 | 0.567 | 0.001 |
| VASH1-AS1 | 0.300 | 0.149 | 0.603 | 0.001 |
| AC015813.1 | 0.714 | 0.562 | 0.907 | 0.006 |
| AC068473.5 | 0.596 | 0.434 | 0.817 | 0.001 |
| LINC01089 | 0.668 | 0.537 | 0.831 | 0.000 |
| USP27X-AS1 | 0.523 | 0.322 | 0.848 | 0.009 |
| AC027575.2 | 0.573 | 0.398 | 0.826 | 0.003 |
| AC106795.2 | 0.758 | 0.627 | 0.917 | 0.004 |
| AC092171.3 | 0.587 | 0.396 | 0.870 | 0.008 |
| AC087500.1 | 0.216 | 0.091 | 0.510 | <0.001 |
| MAN1B1-DT | 0.580 | 0.396 | 0.848 | 0.005 |
| AC074032.1 | 0.477 | 0.293 | 0.774 | 0.003 |
| AC008438.1 | 0.584 | 0.399 | 0.854 | 0.006 |
| AC012640.2 | 0.598 | 0.422 | 0.848 | 0.004 |
| LINC00526 | 0.610 | 0.437 | 0.852 | 0.004 |
| AL118558.3 | 0.578 | 0.423 | 0.792 | 0.001 |
| AC007541.1 | 0.303 | 0.143 | 0.642 | 0.002 |
| AC008124.1 | 0.572 | 0.384 | 0.854 | 0.006 |
| AC008443.4 | 0.672 | 0.515 | 0.876 | 0.003 |
| STAG3L5P-PVRIG2P-PILRB | 0.502 | 0.307 | 0.821 | 0.006 |
| LINC00957 | 0.596 | 0.427 | 0.832 | 0.002 |
| AL121583.1 | 0.304 | 0.158 | 0.586 | <0.001 |
| AC124016.2 | 0.581 | 0.405 | 0.832 | 0.003 |
| AL356740.1 | 0.397 | 0.223 | 0.706 | 0.002 |
| AC023509.4 | 0.561 | 0.391 | 0.806 | 0.002 |
| AL136295.6 | 0.525 | 0.352 | 0.781 | 0.001 |
| AC092171.5 | 0.685 | 0.537 | 0.873 | 0.002 |
| DNAJC3-DT | 0.648 | 0.474 | 0.887 | 0.007 |
| AC068620.2 | 0.168 | 0.072 | 0.390 | 0.000 |
| AC091271.1 | 0.557 | 0.374 | 0.830 | 0.004 |
| AL118558.4 | 0.469 | 0.300 | 0.735 | 0.001 |
| AL121601.1 | 0.451 | 0.253 | 0.806 | 0.007 |
| AC142472.1 | 0.369 | 0.211 | 0.647 | 0.001 |
| AC069224.1 | 0.288 | 0.132 | 0.630 | 0.002 |
| ZSCAN16-AS1 | 0.754 | 0.625 | 0.909 | 0.003 |
| EXOC3-AS1 | 0.719 | 0.573 | 0.903 | 0.005 |
| ATP6V0E2-AS1 | 0.385 | 0.199 | 0.745 | 0.005 |
| AP002360.1 | 0.787 | 0.678 | 0.914 | 0.002 |
| IPO5P1 | 0.570 | 0.409 | 0.793 | 0.001 |
| AC099522.2 | 0.486 | 0.299 | 0.789 | 0.004 |
| AC104986.2 | 0.667 | 0.509 | 0.873 | 0.003 |
| AL022328.4 | 0.303 | 0.164 | 0.560 | 0.000 |
| AC090617.5 | 0.690 | 0.546 | 0.873 | 0.002 |
| Z97653.1 | 0.511 | 0.323 | 0.808 | 0.004 |
| AC026471.2 | 0.269 | 0.120 | 0.606 | 0.002 |
| AC099778.1 | 0.134 | 0.047 | 0.382 | <0.001 |
| SNHG9 | 0.899 | 0.833 | 0.970 | 0.006 |
| NEBL-AS1 | 0.488 | 0.283 | 0.841 | 0.010 |
| AC036176.1 | 0.553 | 0.377 | 0.812 | 0.002 |
| AC005696.4 | 0.629 | 0.455 | 0.871 | 0.005 |
| AL359513.1 | 0.535 | 0.355 | 0.807 | 0.003 |
| U47924.2 | 0.475 | 0.290 | 0.780 | 0.003 |
| PXN-AS1 | 0.586 | 0.398 | 0.865 | 0.007 |
| AC064836.3 | 0.557 | 0.395 | 0.785 | 0.001 |
| AC026979.2 | 0.772 | 0.641 | 0.931 | 0.007 |
| AL355472.1 | 0.742 | 0.609 | 0.903 | 0.003 |
| AC068338.3 | 0.459 | 0.299 | 0.705 | <0.001 |
| AC024075.2 | 0.642 | 0.513 | 0.803 | <0.001 |
| AL691432.2 | 0.764 | 0.640 | 0.912 | 0.003 |
| AL136304.1 | 0.431 | 0.247 | 0.752 | 0.003 |
| FGF14-AS2 | 0.694 | 0.547 | 0.881 | 0.003 |
| LINC02001 | 0.802 | 0.700 | 0.918 | 0.001 |
| AC093249.6 | 0.350 | 0.181 | 0.675 | 0.002 |
| ATP2B1-AS1 | 0.292 | 0.129 | 0.660 | 0.003 |
| AL050341.2 | 0.650 | 0.508 | 0.832 | 0.001 |
| TRAF3IP2-AS1 | 0.100 | 0.027 | 0.366 | 0.001 |
| LOH12CR2 | 0.430 | 0.244 | 0.757 | 0.003 |
| LINC01144 | 0.426 | 0.223 | 0.814 | 0.010 |
| SNHG8 | 0.973 | 0.958 | 0.989 | 0.001 |
| AL133338.1 | 0.403 | 0.213 | 0.762 | 0.005 |
| ZNF667-AS1 | 0.733 | 0.609 | 0.883 | 0.001 |
| ZNF674-AS1 | 0.521 | 0.344 | 0.788 | 0.002 |
| RASSF8-AS1 | 0.490 | 0.310 | 0.774 | 0.002 |
| AC012213.4 | 0.602 | 0.422 | 0.860 | 0.005 |
| AC095057.3 | 0.400 | 0.239 | 0.670 | 0.001 |
| Z95115.1 | 0.582 | 0.398 | 0.850 | 0.005 |
| SNHG19 | 0.968 | 0.946 | 0.990 | 0.005 |
| AC008969.1 | 0.244 | 0.104 | 0.574 | 0.001 |
| AC008669.1 | 0.452 | 0.254 | 0.805 | 0.007 |
| AC079414.3 | 0.457 | 0.264 | 0.793 | 0.005 |
| AC125257.1 | 0.666 | 0.519 | 0.853 | 0.001 |
| AL358472.2 | 0.271 | 0.133 | 0.552 | <0.001 |
| AP000894.4 | 0.689 | 0.534 | 0.888 | 0.004 |
| AC026801.2 | 0.577 | 0.394 | 0.846 | 0.005 |
| AC009812.1 | 0.491 | 0.313 | 0.770 | 0.002 |
| AC007066.2 | 0.194 | 0.079 | 0.473 | 0.000 |
| LINC00663 | 0.189 | 0.071 | 0.504 | 0.001 |
| AL662844.4 | 0.363 | 0.208 | 0.633 | 0.000 |
| AC107375.1 | 0.492 | 0.317 | 0.764 | 0.002 |
| ZNF793-AS1 | 0.617 | 0.447 | 0.850 | 0.003 |
| RNF157-AS1 | 0.585 | 0.404 | 0.848 | 0.005 |
| AC012313.2 | 0.476 | 0.279 | 0.814 | 0.007 |
| AC145207.5 | 0.343 | 0.196 | 0.602 | <0.001 |
| AC020910.4 | 0.668 | 0.513 | 0.870 | 0.003 |
| AL133520.1 | 0.417 | 0.251 | 0.691 | 0.001 |
| AC025181.2 | 0.649 | 0.508 | 0.828 | 0.001 |
| AC092171.2 | 0.692 | 0.558 | 0.858 | 0.001 |
| AC091729.3 | 0.635 | 0.465 | 0.868 | 0.004 |
| LINC01128 | 0.549 | 0.364 | 0.828 | 0.004 |
| AL596244.1 | 0.407 | 0.223 | 0.744 | 0.004 |
| AC073896.3 | 0.420 | 0.232 | 0.759 | 0.004 |
| AC010478.1 | 0.501 | 0.302 | 0.832 | 0.008 |
| AC104825.1 | 0.584 | 0.407 | 0.837 | 0.003 |
| MIR3936HG | 0.594 | 0.415 | 0.851 | 0.005 |
| LINC01963 | 0.641 | 0.495 | 0.829 | 0.001 |
| HNF1A-AS1 | 0.806 | 0.700 | 0.929 | 0.003 |
| AC012313.5 | 0.339 | 0.169 | 0.681 | 0.002 |
| AL162377.1 | 0.401 | 0.228 | 0.705 | 0.001 |
| AP003486.1 | 0.539 | 0.351 | 0.829 | 0.005 |
| TRIM52-AS1 | 0.827 | 0.732 | 0.934 | 0.002 |
| AC254562.3 | 0.376 | 0.204 | 0.693 | 0.002 |
| AL035071.1 | 0.726 | 0.608 | 0.866 | <0.001 |
| AP000757.1 | 0.658 | 0.495 | 0.876 | 0.004 |
| LINC00909 | 0.516 | 0.354 | 0.752 | 0.001 |
| TRPC7-AS1 | 0.528 | 0.333 | 0.837 | 0.007 |
| AP001486.2 | 0.364 | 0.172 | 0.774 | 0.009 |
| AP000892.2 | 0.533 | 0.352 | 0.808 | 0.003 |
| PSMG3-AS1 | 0.447 | 0.273 | 0.734 | 0.001 |
| AC025175.1 | 0.307 | 0.152 | 0.620 | 0.001 |
| MHENCR | 0.837 | 0.754 | 0.929 | 0.001 |
| SNHG20 | 0.564 | 0.406 | 0.784 | 0.001 |
| AC012615.1 | 0.615 | 0.473 | 0.800 | <0.001 |
| AL162274.2 | 0.563 | 0.375 | 0.844 | 0.005 |
| AL136295.7 | 0.643 | 0.485 | 0.852 | 0.002 |
| PTOV1-AS2 | 0.743 | 0.628 | 0.879 | 0.001 |
| AC107952.2 | 0.578 | 0.401 | 0.833 | 0.003 |
| TOB1-AS1 | 0.374 | 0.206 | 0.680 | 0.001 |
| FLNB-AS1 | 1.342 | 1.080 | 1.667 | 0.008 |
| AC008966.1 | 0.436 | 0.235 | 0.808 | 0.008 |
| AC009779.2 | 0.749 | 0.627 | 0.894 | 0.001 |
| AL354920.1 | 0.641 | 0.465 | 0.883 | 0.007 |
| OLMALINC | 0.741 | 0.597 | 0.921 | 0.007 |
| AL359091.4 | 0.333 | 0.151 | 0.734 | 0.006 |
| AC006449.6 | 0.448 | 0.280 | 0.717 | 0.001 |
| AC099850.3 | 1.311 | 1.140 | 1.508 | <0.001 |
| AL049555.1 | 1.218 | 1.072 | 1.385 | 0.003 |
| AL451042.2 | 1.607 | 1.193 | 2.165 | 0.002 |
| CAMTA1-DT | 0.492 | 0.288 | 0.842 | 0.010 |
| AC011445.2 | 0.715 | 0.558 | 0.917 | 0.008 |
| AC005696.1 | 0.383 | 0.222 | 0.660 | 0.001 |
| AC104564.3 | 0.590 | 0.398 | 0.875 | 0.009 |
| AL513165.1 | 0.707 | 0.583 | 0.858 | <0.001 |
| MIR193BHG | 1.450 | 1.184 | 1.776 | <0.001 |
| AP001625.2 | 0.593 | 0.411 | 0.856 | 0.005 |
| AL137779.2 | 0.303 | 0.148 | 0.619 | 0.001 |
| SCAMP1-AS1 | 0.707 | 0.573 | 0.873 | 0.001 |

Supplementary Table 3. LncRNA-mRNA co-expression data.

| lncRNA | mRNA | cor | pvalue |
| --- | --- | --- | --- |
| AC068620.2 | PIK3CA | -0.445 | <0.001 |
| AC068620.2 | NRAS | -0.471 | <0.001 |
| AC068620.2 | HRAS | 0.504 | <0.001 |
| AC068620.2 | KEAP1 | 0.436 | <0.001 |
| AC068620.2 | PHKG2 | 0.420 | <0.001 |
| AC068620.2 | ULK1 | 0.571 | <0.001 |
| AC068620.2 | ATG4D | 0.612 | <0.001 |
| AC068620.2 | MAP1LC3A | 0.513 | <0.001 |
| AC068620.2 | GABARAPL2 | 0.432 | <0.001 |
| AC068620.2 | WIPI2 | 0.528 | <0.001 |
| AC068620.2 | PEBP1 | 0.440 | <0.001 |
| AC068620.2 | CDO1 | 0.505 | <0.001 |
| AC068620.2 | ANO6 | -0.537 | <0.001 |
| AC068620.2 | BACH1 | -0.472 | <0.001 |
| AC068620.2 | LONP1 | 0.653 | <0.001 |
| AC068620.2 | VLDLR | 0.408 | <0.001 |
| AC068620.2 | RGS4 | 0.437 | <0.001 |
| AC068620.2 | GPX4 | 0.563 | <0.001 |
| AC068620.2 | NFE2L2 | -0.427 | <0.001 |
| AC068620.2 | SLC2A8 | 0.508 | <0.001 |
| AC068620.2 | SP1 | -0.416 | <0.001 |
| AC068620.2 | NFS1 | 0.548 | <0.001 |
| AC092171.2 | HRAS | 0.430 | <0.001 |
| AC092171.2 | MAP1LC3A | 0.406 | <0.001 |
| AC092171.2 | WIPI2 | 0.615 | <0.001 |
| AC092171.2 | ATG13 | 0.407 | <0.001 |
| AC092171.2 | PRKAA1 | -0.468 | <0.001 |
| AC092171.2 | HSD17B11 | -0.454 | <0.001 |
| AC092171.2 | SP1 | -0.444 | <0.001 |
| AC092171.2 | NF2 | 0.547 | <0.001 |
| AC099850.3 | PIK3CA | 0.402 | <0.001 |
| AC099850.3 | NRAS | 0.449 | <0.001 |
| AC099850.3 | SLC38A1 | 0.439 | <0.001 |
| AC099850.3 | EGFR | 0.411 | <0.001 |
| AC099850.3 | PEBP1 | -0.456 | <0.001 |
| AC099850.3 | MTDH | 0.431 | <0.001 |
| AC099850.3 | PANX1 | 0.410 | <0.001 |
| AC099850.3 | EIF2S1 | 0.499 | <0.001 |
| AC099850.3 | RRM2 | 0.634 | <0.001 |
| AC099850.3 | AURKA | 0.683 | <0.001 |
| AC099850.3 | FANCD2 | 0.568 | <0.001 |
| AC099850.3 | ISCU | -0.431 | <0.001 |
| AC099850.3 | ACSL3 | 0.405 | <0.001 |
| LINC01705 | NOX4 | 0.625 | <0.001 |
| LINC01705 | PANX1 | 0.468 | <0.001 |
| MIR193BHG | PANX1 | 0.403 | <0.001 |
| MIR193BHG | STEAP3 | 0.455 | <0.001 |
| MIR193BHG | SLC2A1 | 0.443 | <0.001 |
| MIR193BHG | CAV1 | 0.633 | <0.001 |
| TRAF3IP2-AS1 | ACSF2 | -0.454 | <0.001 |
| TRAF3IP2-AS1 | GOT1 | 0.405 | <0.001 |
| TRAF3IP2-AS1 | MAP1LC3A | 0.431 | <0.001 |
| TRAF3IP2-AS1 | GABARAPL2 | 0.677 | <0.001 |
| TRAF3IP2-AS1 | ATG16L1 | -0.458 | <0.001 |
| TRAF3IP2-AS1 | ULK2 | 0.620 | <0.001 |
| TRAF3IP2-AS1 | DPP4 | 0.630 | <0.001 |
| TRAF3IP2-AS1 | PEBP1 | 0.665 | <0.001 |
| TRAF3IP2-AS1 | SLC1A4 | 0.525 | <0.001 |
| TRAF3IP2-AS1 | VLDLR | 0.488 | <0.001 |
| TRAF3IP2-AS1 | SLC3A2 | 0.460 | <0.001 |
| TRAF3IP2-AS1 | RGS4 | 0.486 | <0.001 |
| TRAF3IP2-AS1 | RELA | -0.488 | <0.001 |
| TRAF3IP2-AS1 | STMN1 | 0.450 | <0.001 |
| TRAF3IP2-AS1 | CAPG | -0.442 | <0.001 |
| TRAF3IP2-AS1 | YWHAE | 0.686 | <0.001 |
| TRAF3IP2-AS1 | NQO1 | -0.421 | <0.001 |
| TRAF3IP2-AS1 | MUC1 | -0.456 | <0.001 |
| TRAF3IP2-AS1 | CISD1 | 0.502 | <0.001 |
| TRAF3IP2-AS1 | FADS2 | 0.591 | <0.001 |
| TRAF3IP2-AS1 | SRC | -0.467 | <0.001 |
| TRAF3IP2-AS1 | PML | -0.439 | <0.001 |
| TRAF3IP2-AS1 | ENPP2 | 0.466 | <0.001 |
| TRAF3IP2-AS1 | ISCU | 0.559 | <0.001 |
| TRAF3IP2-AS1 | PROM2 | -0.452 | <0.001 |
| TRAF3IP2-AS1 | GCH1 | 0.739 | <0.001 |
